# Comparative Analysis of Diabetic Ketoacidosis in Adults With Type 1 and Type 2 Diabetes Mellitus: Insights From a Saudi Arabian Cohort

**DOI:** 10.1155/jobe/3964619

**Published:** 2025-03-26

**Authors:** Sadiq A. Alali, Saqib A. Ghulam, Khlood A. Bukhamsin, Khadijah Al Nas, Aliaa Alhashim, Danna AlMoaber, Maryam Al-Khalifah, Ebtehal Almarzooq, Alzhra H. Albin Alshaikh, Sadiq M. AlHowdar, Bader A. Alhammad

**Affiliations:** ^1^Diabetologist Consultant, King Fahad Hospital, Al Hofuf, Eastern Province, Saudi Arabia; ^2^Endocrinologist Consultant, King Salman Hospital, Riyadh, Riyadh Province, Saudi Arabia; ^3^Diabetologist Consultant, Eastern Health Cluster, Dammam, Eastern Province, Saudi Arabia; ^4^Diabetologist Consultant, King Salman Hospital, Riyadh, Riyadh Province, Saudi Arabia; ^5^Family Medicine Consultant, Al-Ahsa Health Cluster, Al Hofuf, Eastern Province, Saudi Arabia; ^6^King Faisal University College of Medicine, Al Ahsa, Eastern Province, Saudi Arabia; ^7^King Fahad Hospital, Al Hofuf, Eastern Province, Saudi Arabia

## Abstract

**Background:** Diabetic ketoacidosis (DKA) is a life-threatening complication commonly seen in Type 1 diabetes mellitus (T1DM) but also affects Type 2 diabetes mellitus (T2DM).

**Objectives:** To compare the clinical presentation, biochemical parameters, and precipitating factors of DKA in adult patients with T1DM and T2DM.

**Methodology:** This retrospective cohort study was conducted at King Salman Hospital, Riyadh, involving medical records of diabetic patients aged 14 years or older who attended the Diabetic Center from September 1, 2021, to August 1, 2022. Data collection included sociodemographic, clinical, biochemical, and management details using a standardized checklist.

**Results:** The study included 285 patients with DKA, aged 14–70 years (mean: 23.1 ± 11.5 years), with 52.5% being male. The most common symptoms were nausea (91.1%), abdominal pain (86.1%), vomiting (83.6%), polyuria/polydipsia (74.1%), and shortness of breath (72.4%). Vomiting and abdominal pain were more frequent in T1DM (85.9% and 88.3%) compared to T2DM (65.6% and 68.8%), *p*=0.004 and 0.003, respectively, while dizziness was more common in T2DM (56.3% vs. 33.2%), *p*=0.011. Uric acid and creatinine levels were significantly higher in T2DM, whereas hemoglobin and hematocrit were elevated in T1DM. Poor compliance was the most common precipitating factor (70.2%), followed by upper respiratory tract infection (21.1%) and inadequate treatment (15.6%).

**Conclusion:** This study highlights key differences in DKA presentation between T1DM and T2DM. While symptoms such as nausea and abdominal pain were common in both types, vomiting was more frequent in T1DM and dizziness in T2DM. Biochemical markers such as uric acid and creatinine were elevated in T2DM, while hemoglobin and hematocrit were higher in T1DM. Poor compliance was a more common precipitating factor in T1DM, whereas inadequate treatment prevailed in T2DM. Tailored management approaches for each diabetes type may improve DKA outcomes.

## 1. Introduction

Diabetes mellitus (DM) is considered one of the most critical global health emergencies of the 21st century, currently affecting approximately 415 million adults worldwide aged 20–79 years [1, 2].

In the Kingdom of Saudi Arabia (KSA), Type 2 DM (T2DM) has one of the highest prevalence rates globally, attributed to a combination of factors such as rapid urbanization, sedentary lifestyles, genetic predisposition, and a shift towards high-calorie, low-nutrient diets [3]. Recent research suggests that more than 44% of individuals aged 55 or older in Saudi Arabia have severe or uncontrolled diabetes with long-term complications [4].

Type 1 DM (T1DM) is also prevalent, particularly among children and adolescents, in Saudi Arabia and worldwide [5]. It is a chronic disease marked by hyperglycemia due to insulin deficiency, which is a primary cause of diabetic ketoacidosis (DKA). In DKA, insufficient insulin levels lead to elevated blood sugar and trigger metabolic responses, including increased lipolysis and the release of free fatty acids, which are subsequently oxidized into ketone bodies, acetoacetate, beta-hydroxybutyrate, and acetone, that accumulate and lead to metabolic acidosis [6]. More in-depth discussions on the pathophysiological mechanisms connecting hyperglycemia to DKA are available in studies by Kitabchi et al. and Savage et al. [7, 8]. According to the Diabetes Atlas (eighth edition), approximately 35,000 children and adolescents in Saudi Arabia are affected by T1DM, making the country eighth globally in total T1DM cases and fourth in annual incidence, with a rate of 31.4 per 100,000 young individuals [9, 10].

Historically, DKA has been viewed as a complication primarily associated with T1DM. However, it is increasingly observed among individuals with T2DM, reflecting an evolving understanding of diabetes complications. In T2DM, DKA typically emerges at advanced stages, resulting from a complex interplay between beta-cell dysfunction, leading to relative or absolute insulin deficiency, and the presence of insulin resistance. This dynamic exacerbates hyperglycemia and ketosis, precipitating the onset of DKA [11]. The prevalence of DKA varies worldwide, influenced by regional and healthcare disparities [12]. Understanding the mechanisms underlying DKA development in T2DM, particularly the role of insulin resistance, is essential for effective management and improved patient outcomes.

In Western countries, the annual incidence of DKA ranges from 56 to 128 per 1000 patients with T1DM [13]. DKA–related mortality is significantly higher in developing countries (6%–24%) compared to Western countries (0.15%–0.31%) [14, 15].

Several studies have investigated the differences in DKA presentation between T1DM and T2DM, focusing on clinical and biochemical characteristics, symptomatology, severity of metabolic disturbances, treatment response, and associated complications. Understanding these distinctions is crucial for accurate diagnosis, timely intervention, and the formulation of tailored management strategies that enhance patient outcomes. By exploring the unique characteristics of DKA in T1DM and T2DM, healthcare providers can better address the specific needs of each patient group [16–18]. However, to the best of our knowledge, no studies have examined these comparative characteristics of DKA between T1DM and T2DM patients in Saudi Arabia.

This study aims to explore the clinical and biochemical parameters, as well as the severity, of DKA among adults attending the Diabetic Center at King Salman Hospital, Riyadh, Saudi Arabia.

## 2. Materials and Methods

This retrospective cohort study was conducted at the Diabetic Center, King Salman Hospital, in Riyadh, Saudi Arabia. Riyadh, the largest city and capital, has an estimated population of 7.6 million as of the 2019 census [17].

The study reviewed records of all diabetic patients aged 14 years or older with DKA who attended the Diabetic Center at King Salman Hospital between September 1, 2021, and August 1, 2022. Only patients with Type 1 or Type 2 diabetes were included, and cases of gestational diabetes were excluded. DKA was defined following the American Diabetes Association (ADA) guidelines: blood glucose > 250 mg/dL, blood pH < 7.3, and the presence of ketonemia or ketonuria [19].

Data collection was performed using a structured checklist, compiling information from patient interviews, hospital medical records, and electronic files. The checklist included the following:

• Sociodemographic data: Age at presentation, gender, marital status, and nationality.
• Clinical data: Duration of hospital admission, place of admission, diabetes type, age at diabetes diagnosis, history of hyperglycemic emergencies in the past 3 months, family history of diabetes, and history of hypoglycemia.
• Biochemical data: Glucose (mmol/L), pH, HCO_3_ (mmol/L), sodium (Na, mmol/L), pCO_2_ (mmHg), potassium (K, mmol/L), chloride (Cl, mmol/L), magnesium (Mg, mmol/L), uric acid (μmol/L), anion gap (mmol/L), serum osmolarity (mOsm/kg), urea nitrogen (mmol/L), creatinine (μmol/L), serum ketones (mmol/L), white blood cells (10^9^/L), hemoglobin (g/dL), hematocrit (%), platelets (10^9^/L), urine analysis, glycated hemoglobin (HbA1c, %), thyroid-stimulating hormone (TSH) (mIU/L), and serum lactate levels (mmol/L).

The definitions for precipitating factors are as follows:

• First presentation: The initial diagnosis of diabetes with DKA as the presenting condition.
• Inadequate treatment: Insufficient or inappropriate diabetes management before the DKA episode, including incorrect dosing or medication type.
• Poor compliance: Nonadherence to prescribed diabetes regimens, including missed medication doses or disregarded dietary recommendations.
• Upper respiratory tract infection (URTI): Any upper respiratory infection that may exacerbate blood glucose, contributing to DKA.
• Urinary tract infection (UTI): Any UTI potentially causing stress that leads to DKA.
• Diabetic foot: Foot ulcers or infections related to diabetes that may precipitate DKA.
• Other factors: Conditions such as increased carbohydrate intake, trauma, or other potential DKA triggers.

### 2.1. Statistical Analysis

Data were entered and analyzed using the Statistical Package for Social Sciences (SPSS) software, Version 28.0. Data verification was performed manually before computerized entry, and descriptive statistics (e.g., number, percentage, mean, range, and standard deviation [SD]) were calculated for all variables. The Shapiro–Wilk test was used to assess data normality. For normally distributed continuous variables, the *t*-test compared means between groups. For non-normally distributed continuous variables, the Mann–Whitney *U* test was applied. Chi-square or Fisher Exact tests compared categorical variables. *p* values of ≤ 0.05 were considered statistically significant. SDs were calculated for all variables to provide descriptive insights, even when nonparametric tests were used.

### 2.2. Ethical Considerations

All required official approvals were obtained, including approval from the regional Research and Ethics Committee (Institutional Review Board [“IRB”]) and permissions from the director of the Diabetic Center and the head of the endocrinology unit at King Salman Hospital, Riyadh.

## 3. Results

### 3.1. Sociodemographic Characteristics

The study included 285 diabetic patients with a history of DKA. As summarized in Table 1, 52.5% of the patients were male (*n* = 149) and 47.5% were female (*n* = 135). Patients' ages ranged from 14 to 70 years, with a mean age of 23.1 years and a SD of 11.5 years. The majority of participants were Saudi nationals (89.5%; *n* = 255), and most were unmarried (78.1%; *n* = 221).

### 3.2. Clinical Characteristics

The majority of patients had Type 1 diabetes (88.8%; *n* = 253). Most had been living with diabetes for more than 10 years (61.5%; *n* = 150). The majority reported no recent hyperglycemic emergencies, although 10.7% (*n* = 28) experienced three or more episodes in the past 3 months. A family history of diabetes was present in 33.9% of patients (*n* = 78). Comorbid conditions were reported in 20.4% of patients (*n* = 58), while only 1.1% (*n* = 3) had a history of hypoglycemia. In terms of hospital admission, most patients were admitted either to a general ward (46.6%; *n* = 132) or the emergency room (42.8%; *n* = 121), as detailed in Table 2.

### 3.3. Clinical Presentation

**Figure 1:**
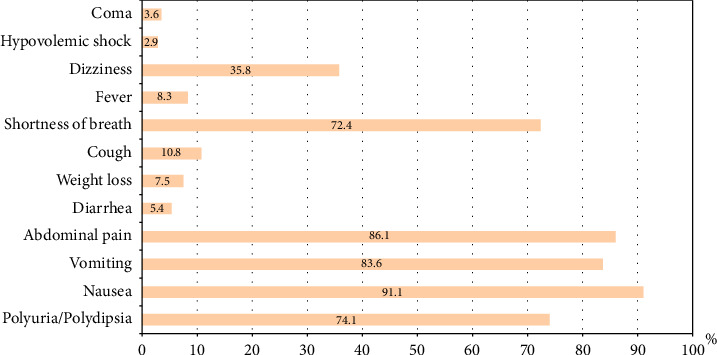
Clinical presentation of cases of diabetic ketoacidosis.

As summarized in Figure 1, the most frequently reported symptoms of DKA were nausea (91.1%; *n* = 255), abdominal pain (86.1%; *n* = 241), vomiting (83.6%; *n* = 235), polyuria/polydipsia (74.1%; *n* = 200), and shortness of breath (72.4%; *n* = 202).

Vomiting and abdominal pain were reported more frequently among patients with Type 1 diabetes compared to those with Type 2 diabetes (85.9%; *n* = 214 and 88.3%; *n* = 218, respectively, vs. 65.6%; *n* = 21 and 68.8%; *n* = 22), with *p* values of 0.004 and 0.003, respectively. Conversely, dizziness was reported more frequently in patients with Type 2 diabetes than in those with Type 1 diabetes (56.3%; *n* = 18 vs. 33.2%; *n* = 82), *p*=0.011 (Table 3).

### 3.4. Clinical Signs

The majority of cases (91.8%; *n* = 246) exhibited acidotic breathing, while 17.7% (*n* = 46) presented with drowsiness. Pulse rate, systolic blood pressure, diastolic blood pressure, temperature, and oxygen saturation values are summarized in Table 4, with each variable presented as a range, arithmetic mean, and standard deviation.

### 3.5. Clinical Sign Comparison

Acidotic breathing was more frequently reported in patients with Type 1 diabetes than in those with Type 2 diabetes (94.1%; *n* = 224 vs. 73.3%; *n* = 22), with a statistically significant *p* value of < 0.001. Both systolic and diastolic blood pressures were higher in patients with Type 2 diabetes compared to those with Type 1 diabetes (137.5 ± 21.7 mmHg and 79.1 ± 17.2 mmHg vs. 121.5 ± 17.0 mmHg and 72.5 ± 13.2 mmHg, with *p* values of < 0.001 and 0.015, respectively) (Table 5).

### 3.6. Self-Monitoring Practices

Patients with Type 1 diabetes were more likely than those with Type 2 diabetes to perform self-blood glucose testing (91.5%; *n* = 225 vs. 79.2%; *n* = 19), although this difference was borderline insignificant (*p*=0.051). In addition, 18.3% (*n* = 43) of patients with Type 1 diabetes performed blood ketone testing, compared to none of the patients with Type 2 diabetes, a statistically significant difference (*p*=0.018) (Table 6).

### 3.7. Follow-Up Practice in the Last 12 Months

**Figure 2:**
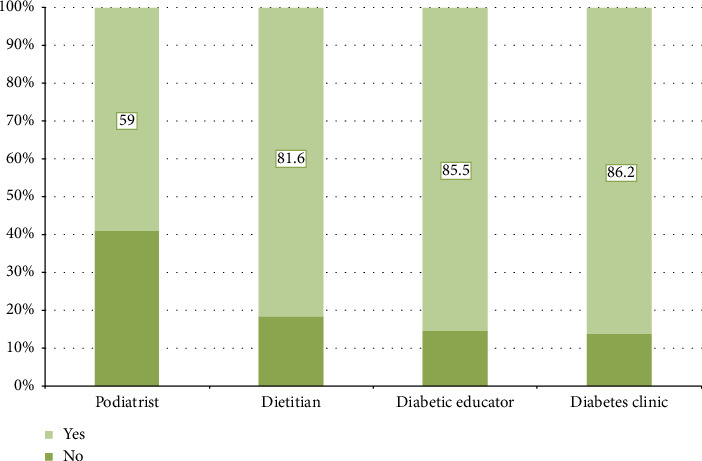
Follow-up practice in the last 12 months among patients with diabetic ketoacidosis.

As shown in Figure 2, 86.2% (*n* = 219) of patients with DKA attended follow-up appointments at a diabetes clinic, while 85.5% (*n* = 218) consulted a diabetic educator, 81.6% (*n* = 209) met with a dietitian, and 59% (*n* = 135) visited a podiatrist within the past 12 months.

### 3.8. Dietitian Follow-Up

Type 1 diabetic patients were more likely than Type 2 diabetic patients to have followed up with a dietitian in the last 12 months (83.1%; *n* = 196 vs. 65.0%; *n* = 13), with this difference reaching statistical significance (*p*=0.045) (Table 7).

### 3.9. Diabetes Management

The majority of patients with DKA (96.6%; *n* = 257) were on diabetes treatment. Most of these patients (75.1%; *n* = 187) were on basal-bolus insulin alone, while 6.0% (*n* = 15) were using oral hypoglycemic medications exclusively, and 2.8% (*n* = 7) were on both insulin and oral hypoglycemic tablets (Table 8).

### 3.10. Biochemical Markers

Uric acid levels were significantly higher in patients with Type 2 diabetes compared to those with Type 1 diabetes (558.70 ± 28.71 vs. 374.90 ± 141.64 μmol/L, *p*=0.001). Similarly, creatinine levels were significantly elevated in Type 2 diabetic patients compared to Type 1 diabetic patients (124.57 ± 43.29 vs. 105.45 ± 38.87 μmol/L, *p*=0.020). These findings likely reflect dehydration, renal impairment, or underlying nephropathy in Type 2 diabetes patients. However, the absence of kidney imaging or complementary inflammatory markers limits definitive conclusions regarding nephropathy or systemic inflammation. Future studies incorporating eGFR and inflammatory markers such as CRP and IL-6 are warranted to strengthen these associations. Conversely, hemoglobin levels were significantly higher in Type 1 diabetic patients than in those with Type 2 diabetes (15.21 ± 10.43 vs. 13.21 ± 2.33 g/dL, *p*=0.017). Hematocrit values also showed a significant increase in Type 1 diabetic patients compared to Type 2 diabetic patients (43.84 ± 5.34% vs. 39.44 ± 5.62%, *p* < 0.001) (Table 9).

### 3.11. Precipitating Factors

**Figure 3:**
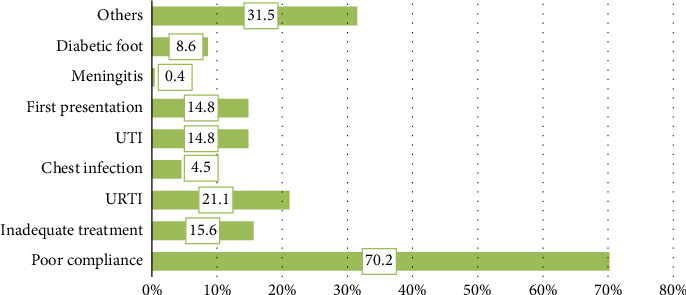
Precipitating factors for diabetic ketoacidosis. UTI: urinary tract infection and URTI: upper respiratory tract infection.

As shown in Figure 3, poor compliance emerged as the most frequently reported precipitating factor (70.2%; *n* = 186), followed by URTI (21.1%; *n* = 55) and inadequate treatment (15.6%; *n* = 41). Poor compliance was significantly more common in Type 1 diabetic patients than in Type 2 diabetic patients (71.9%; *n* = 174 vs. 52.2%; *n* = 12, *p*=0.048). In contrast, inadequate treatment was more prevalent in Type 2 diabetic patients (45.8%; *n* = 11 vs. 12.6%; *n* = 30, *p* < 0.001).

The predominance of poor compliance as a DKA trigger in this cohort contrasts with global findings, where infections are often the leading cause. This discrepancy may reflect regional factors such as differences in healthcare accessibility, cultural practices, and patient education regarding diabetes management. Although infections, including UTIs (33.3%; *n* = 8) and first presentations (33.3%; *n* = 8), were still noted more frequently in Type 2 diabetes patients than in Type 1 diabetes patients (13.0%; *n* = 31 and 12.9%; *n* = 31, respectively, *p*=0.007), they were not as dominant as seen globally. Diabetic foot complications were also significantly higher in Type 2 diabetes patients (35.5%; *n* = 11) compared to Type 1 diabetes patients (5.2%; *n* = 13, *p* < 0.001).

Other factors, including increased carbohydrate intake, trauma, and miscellaneous causes, were more common in Type 1 diabetes patients (33.3%; *n* = 82) than in Type 2 diabetes patients (14.8%; *n* = 4, *p*=0.035). These findings highlight the need for a region-specific approach to DKA prevention, focusing on improving treatment adherence, education, and access to healthcare services (Table 10).

### 3.12. DKA Management and Outcomes

The majority of patients with DKA (98.9%; *n* = 278) began fluid replacement within 1 h of presentation, while 61.1% (*n* = 168) received 5 or fewer units of insulin per kg per hour. Antibiotics were administered to 60.9% (*n* = 170) of patients, and 43.1% (*n* = 121) received enoxaparin. Most patients (91.0%; *n* = 234) were discharged, with no recorded deaths. In addition, 90.0% (*n* = 217) of the patients were rehydrated within 6 h, and 93.9% (*n* = 246) resolved ketosis within 24 h. More than half of the patients (54.7%; *n* = 139) were discharged from the hospital within the first 24 h, while only 6.7% (*n* = 17) remained hospitalized for over 72 h (Table 11).

### 3.13. Insulin and Enoxaparin Administration

The majority of Type 2 diabetic patients (86.2%; *n* = 25) received more than 5 units of insulin per kg per hour, compared to 33.3% (*n* = 82) of Type 1 diabetic patients (*p* < 0.001). In addition, enoxaparin was administered to a significantly larger proportion of Type 2 diabetic patients (84.4%; *n* = 27) compared to Type 1 diabetic patients (37.8%; *n* = 94, *p* < 0.001) (Table 12).

## 4. Discussion

DKA is a serious complication of diabetes with significant morbidity. This study compared clinical characteristics, biochemical markers, precipitating factors, and management of DKA between adults with T1DM and T2DM in a Saudi Arabian cohort, highlighting distinct patterns and challenges specific to this population.

In agreement with global studies [15, 16], this study found that DKA was significantly more common in T1DM patients, consistent with the absolute insulin deficiency characteristic of T1DM that predisposes these patients to ketosis and DKA. However, the presence of DKA in T2DM patients underscores the complexity of this condition, particularly in advanced disease stages involving beta-cell dysfunction and insulin resistance.

Nausea, abdominal pain, vomiting, polyuria/polydipsia, and shortness of breath were the most common presenting symptoms in this cohort. Vomiting and abdominal pain were significantly more frequent in T1DM patients, while dizziness was more prevalent in T2DM patients. The finding of dizziness in T2DM patients warrants further exploration, as it may reflect underlying comorbidities, volume depletion, or other systemic factors. Acidotic breathing was a hallmark of DKA presentation, particularly in T1DM patients, consistent with more pronounced metabolic derangements in this group. Conversely, systolic and diastolic blood pressures were significantly higher among T2DM patients, likely reflecting a higher prevalence of hypertension and age-related comorbidities in this group.

Biochemically, elevated uric acid and creatinine levels were observed in T2DM patients, consistent with dehydration, renal impairment, or nephropathy [20, 21]. However, the lack of complementary kidney function assessments (e.g., eGFR and imaging) and inflammatory markers (e.g., CRP and IL-6) limits our ability to definitively attribute these findings to nephropathy or systemic inflammation. Future prospective studies should address this gap. Elevated hemoglobin and hematocrit levels in T1DM patients may reflect more severe dehydration at presentation.

Precipitating factors for DKA in this cohort diverged from global patterns. Poor compliance was the most common trigger (70.2%), particularly in T1DM patients. This finding contrasts with global studies where infections are frequently cited as the leading cause [22, 23]. The lower frequency of infections in this study (21.1%) may reflect improved infection control practices or underreporting due to the retrospective nature of the study. Inadequate treatment, noted more commonly in T2DM patients, emphasizes the importance of addressing gaps in treatment adherence and healthcare accessibility. These regional differences highlight the need for context-specific interventions, including patient education and improvements in healthcare delivery systems.

The management of DKA in this cohort was effective, with most patients achieving ketosis resolution within 24 h and being discharged without dehydration. T2DM patients required higher insulin doses and enoxaparin, reflecting their distinct metabolic and thromboembolic risks. These findings reinforce the importance of individualized management strategies tailored to diabetes type and patient-specific factors.

## 5. Limitations

This study has several limitations. Its retrospective design, relying on medical record accuracy and completeness, may have introduced reporting biases. The single-center scope limits the generalizability of findings to the broader Saudi population or other regions with differing healthcare systems. Expanding future research to include multicenter cohorts is essential to enhance representativeness. The lack of kidney imaging and inflammatory markers limits the ability to conclusively link uric acid and creatinine elevations to nephropathy or systemic inflammation. Ethical transparency also requires clarification; while IRB approval was obtained, the study did not explicitly state whether patient consent was waived or obtained. Future studies should address these gaps through prospective designs, broader scopes, and explicit ethical disclosures.

## 6. Conclusion

This study highlights distinct differences between T1DM and T2DM in the presentation, biochemical markers, precipitating factors, and management of DKA in a Saudi Arabian cohort. While nausea, vomiting, and abdominal pain were common across both types, vomiting was more prevalent in T1DM patients, and dizziness was more frequent in T2DM patients. Biochemically, T2DM patients exhibited higher uric acid and creatinine levels, while hemoglobin and hematocrit were elevated in T1DM patients. Poor compliance emerged as the leading precipitating factor in T1DM, whereas inadequate treatment was more prevalent in T2DM.

Management strategies demonstrated effective resolution of ketosis and dehydration within 24 h for most patients. However, the higher use of insulin and enoxaparin in T2DM patients highlights their distinct therapeutic needs.

These findings emphasize the importance of tailoring DKA prevention and management strategies to the specific needs of T1DM and T2DM patients. Addressing regional factors, including healthcare access, patient education, and cultural influences, is critical for reducing DKA incidence. Future studies should incorporate multicenter, prospective designs and include comprehensive biochemical and inflammatory markers to validate and expand upon these findings.

## Figures and Tables

**Table 1:** Sociodemographic characteristics of the patients (*n* = 285).

Variables	Frequency	Percentage (%)
Gender (*n* = 284)		
Male	149	52.5
Female	135	47.5
Age at presentation (years)		
Range	14–70	
Mean ± SD⁣^∗^	23.1 ± 11.5	
Nationality		
Saudi	255	89.5
Non-Saudi	30	10.5
Marital status (*n* = 283)		
Unmarried	221	78.1
Married	62	21.9

⁣^∗^SD, standard deviation.

**Table 2:** Clinical characteristics of the patients (*n* = 285).

Variables	Frequency	Percentage (%)
Type of diabetes		
Type 1	253	88.8
Type 2	32	11.2
Duration of diabetes (years) (*n* = 244)		
≤ 5	25	10.2
6–10	69	28.3
> 10	150	61.5
Number of hyperglycemic emergencies in last 3 months (*n* = 261)		
No	160	61.3
Once	53	20.3
Twice	20	7.7
3 times or more	28	10.7
Family history of diabetes (*n* = 230)		
No	145	63.0
Yes, father	30	13.0
Yes, mother	13	5.7
Yes, both	28	12.2
Yes, siblings	17	7.4
Yes, children	1	0.5
Do not know	7	3.0
History of other comorbidities		
No	227	79.6
Yes	58	20.4
History of hypoglycemia		
No	282	98.9
Yes	3	1.1
Place of admission (*n* = 283)		
Ward	132	46.6
ER⁣^∗^	121	42.8
Both	18	6.4
ICU⁣^∗∗^	6	2.1
Ward and ICU	4	1.4
Ward, ER, and ICU	2	0.7

⁣^∗^ER, emergency room.

⁣^∗∗^ICU, intensive care unit.

**Table 3:** Comparison of clinical presentation of diabetic ketoacidosis between Type 1 and Type 2 diabetes mellitus patients.

Symptom	Type 1 diabetes *N* (%)	Type 2 diabetes *N* (%)	*p* value⁣^∗^
Polyuria/polydipsia (*n* = 270)	174 (73.1)	26 (81.3)	0.324
Nausea (*n* = 280)	228 (91.6)	27 (87.1)	0.294
Vomiting (*n* = 281)	214 (85.9)	21 (65.6)	0.004
Abdominal pain (*n* = 280)	219 (88.3)	22 (68.8)	0.003
Diarrhea (*n* = 278)	14 (5.7)	1 (3.1)	0.484
Weight loss (*n* = 279)	17 (6.9)	4 (12.9)	0.192
Cough (*n* = 278)	28 (11.3)	2 (6.5)	0.320
Shortness of breath (*n* = 279)	183 (74.1)	19 (59.4)	0.080
Fever (*n* = 278)	21 (8.5)	2 (6.3)	0.491
Dizziness (*n* = 279)	82 (33.2)	18 (56.3)	0.011
Hypovolemic shock (*n* = 277)	6 (2.4)	2 (6.3)	0.233
Coma (*n* = 278)	7 (2.8)	3 (9.4)	0.095

⁣^∗^Chi-square/Fisher exact tests.

**Table 4:** Clinical presentation of diabetic ketoacidosis cases.

Variable	Frequency	Percentage (%) or range	Mean ± SD
Acidotic breathing (*n* = 268)			
No	22	8.2	
Yes	246	91.8	
Drowsiness (*n* = 260)			
No	214	82.3	
Yes	46	17.7	
Pulse (*n* = 270)		65–149	107.6 ± 18.7
Systolic blood pressure (*n* = 269)		76–195	123.2 ± 18.2
Diastolic blood pressure (*n* = 269)		35–144	73.2 ± 13.8
Temperature (*n* = 268)		36.0–37.8	36.8 ± 0.3
Oxygen saturation % (*n* = 267)		86–100	97.5 ± 1.9

**Table 5:** Comparison of clinical signs between Type 1 and Type 2 diabetes mellitus patients.

Clinical sign	Type 1 diabetes *N* (%)	Type 2 diabetes *N* (%)	*p* value
Acidotic breathing (*n* = 268)			
No (*n* = 22)	14 (5.9)	8 (26.7)	< 0.001⁣^∗^
Yes (*n* = 246)	224 (94.1)	22 (73.3)	
Drowsiness (*n* = 260)			
No (*n* = 214)	193 (83.2)	21 (75.0)	0.283⁣^∗^
Yes (*n* = 46)	39 (16.8)	7 (25.0)	
Pulse (*n* = 270)			
Mean ± SD	108.1 ± 18.7	102.6 ± 18.0	0.137⁣^∗∗^
Systolic blood pressure (*n* = 269)			
Mean ± SD	121.5 ± 17.0	137.5 ± 21.7	< 0.001⁣^∗∗^
Diastolic blood pressure (*n* = 269)			
Mean ± SD	72.5 ± 13.2	79.1 ± 17.2	0.015⁣^∗∗^
Temperature (*n* = 268)			
Mean ± SD	36.8 ± 0.3	36.9 ± 0.4	0.274⁣^∗∗^
Oxygen saturation % (*n* = 267)			
Mean ± SD	97.5 ± 1.9	97.6 ± 1.9	0.851⁣^∗∗^

⁣^∗^Chi-square test.

⁣^∗∗^Independent two-sample *t*-test.

**Table 6:** Comparison of self-care practices between Type 1 and Type 2 diabetes mellitus patients.

Self-care practice	Type 1 diabetes *N* = 246 *N* (%)	Type 2 diabetes *N* = 24 *N* (%)	*p* value⁣^∗^
Blood glucose testing (*n* = 270)			
No (*n* = 26)	21 (8.5)	5 (20.8)	0.051
Yes (*n* = 244)	225 (91.5)	19 (79.2)	
Blood ketone testing (*n* = 256)			
No (*n* = 213)	192 (81.7)	21 (100)	0.018
Yes (*n* = 43)	43 (18.3)	0 (0.0)	

⁣^∗^Chi-square test.

**Table 7:** Comparison of follow-up practice in the last 12 months between Type 1 and Type 2 diabetes mellitus patients.

Follow-up practice	Type 1 diabetes *N* (%)	Type 2 diabetes *N* (%)	*p* value⁣^∗^
Podiatrist (*n* = 229)			
No (*n* = 94)	87 (41.4)	7 (36.8)	0.697
Yes (*n* = 135)	123 (58.6)	12 (63.2)	
Dietitian (*n* = 256)			
No (*n* = 47)	40 (16.9)	7 (35.0)	0.045
Yes (*n* = 209)	196 (83.1)	13 (65.0)	
Diabetic educator (*n* = 255)			
No (*n* = 37)	33 (14.0)	4 (20.0)	0.326⁣^∗∗^
Yes (*n* = 218)	202 (86.0)	16 (80.0)	
Diabetes clinic (*n* = 254)			
No (*n* = 35)	31 (13.2)	4 (20.0)	0.290⁣^∗∗^
Yes (*n* = 219)	203 (86.8)	16 (80.0)	

⁣^∗^Chi-square test.

⁣^∗∗^Fisher's exact test.

**Table 8:** Diabetes management among patients with diabetic ketoacidosis.

Variables	Frequency	Percentage (%)
On treatment (*n* = 266)		
No	9	3.4
Yes	257	96.6
Insulin (*n* = 249)		
Basal-bolus	187	75.1
Premix	39	15.7
CSII⁣^∗^	5	2.0
Others	18	7.2
Oral hypoglycemic tablets (*n* = 250)		
No	235	94.0
Yes	15	6.0
Both insulin and oral hypoglycemic tablets (*n* = 251)		
No	244	97.2
Yes	7	2.8

⁣^∗^CSII, continuous subcutaneous insulin infusion.

**Table 9:** Comparison of biochemical markers between Type 1 and Type 2 diabetic patients with diabetic ketoacidosis.

Variable	*n*	All patients (mean ± SD)	Type 1 diabetes (mean ± SD)	Type 2 diabetes (mean ± SD)	*p* value	Test used
Glucose (mmol/L)	264	30.19 ± 23.42	30.19 ± 24.26	30.18 ± 15.63	0.998	*t*-test
pH	272	7.12 ± 0.14	7.12 ± 0.14	7.11 ± 0.16	0.828	*t*-test
Bicarbonate (mmol/L)	254	23.07 ± 7.75	22.99 ± 7.70	23.73 ± 8.32	0.652	*t*-test
Sodium (mmol/L)	256	132.37 ± 13.95	132.47 ± 14.66	131.55 ± 4.92	0.746	*t*-test
Potassium (mmol/L)	256	5.24 ± 8.08	5.32 ± 8.54	4.55 ± 1.02	0.640	Mann–Whitney U
Chloride (mmol/L)	249	95.43 ± 12.11	95.48 ± 12.63	95.03 ± 6.13	0.860	*t*-test
Magnesium (mmol/L)	81	0.78 ± 0.22	0.77 ± 0.22	0.87 ± 0.19	0.201	Mann–Whitney U
Uric acid (μmol/L)	34	385.71 ± 144.22	374.90 ± 141.64	558.70 ± 28.71	0.001	Mann–Whitney U
Anion gap (mmol/L)	17	24.45 ± 3.37	24.49 ± 2.88	24.36 ± 4.77	0.944	*t*-test
Serum osmolarity (mOsm/kg)	72	289.96 ± 18.46	288.63 ± 14.55	300.63 ± 37.49	0.083	Mann–Whitney U
Urea nitrogen (mmol/L)	248	6.86 ± 7.78	6.76 ± 8.02	7.72 ± 5.37	0.550	Mann–Whitney U
Creatinine (μmol/L)	247	107.46 ± 39.70	105.45 ± 38.87	124.57 ± 43.29	0.020	Mann–Whitney U
Serum ketones (mmol/L)	242	5.29 ± 1.14	5.30 ± 1.09	5.21 ± 1.53	0.712	*t*-test
White blood cells (10^9^/L)	249	15.27 ± 9.51	15.15 ± 9.35	16.22 ± 10.96	0.588	Mann–Whitney U
Hemoglobin (g/dL)	248	15.00 ± 9.91	15.21 ± 10.43	13.21 ± 2.33	0.017	Mann–Whitney U
Hematocrit (%)	243	43.37 ± 5.53	43.84 ± 5.34	39.44 ± 5.62	< 0.001	Mann–Whitney U
Glycosylated hemoglobin (%)	73	11.47 ± 1.86	11.61 ± 1.84	10.90 ± 1.90	0.205	*t*-test
Serum lactate (mmol/L)	60	2.19 ± 1.63	2.22 ± 1.59	2.09 ± 1.82	0.799	Mann–Whitney U

*Note:* Independent two-sample *t*-test/Mann–Whitney *U* test.

Abbreviation: SD, standard deviation.

**Table 10:** Comparison of precipitating factors of diabetic ketoacidosis between Type 1 and Type 2 diabetes mellitus patients.

Precipitating factor	Type 1 diabetes (*n* = 242) *n* (%)	Type 2 diabetes (*n* = 23) *n* (%)	*p* value	Test used
Poor compliance (*n* = 265)				
No (*n* = 79)	68 (28.1)	11 (47.8)	0.048	Chi-square⁣^*∗*^
Yes (*n* = 186)	174 (71.9)	12 (52.2)		
Inadequate treatment (*n* = 263)				
No (*n* = 222)	209 (87.4)	13 (54.2)	< 0.001	Chi-square
Yes (*n* = 41)	30 (12.6)	11 (45.8)		
Upper respiratory tract infection (*n* = 261)				
No (*n* = 206)	184 (77.6)	22 (91.7)	0.082	Fisher's exact⁣^*∗∗*^
Yes (*n* = 55)	53 (22.4)	2 (8.3)		
Urinary tract infection (*n* = 263)				
No (*n* = 224)	208 (87.0)	16 (66.7)	0.007	Fisher's exact
Yes (*n* = 39)	31 (13.0)	8 (33.3)		
First presentation (*n* = 264)				
No (*n* = 225)	209 (87.1)	16 (66.7)	0.007	Fisher's exact
Yes (*n* = 39)	31 (12.9)	8 (33.3)		
Diabetic foot (*n* = 279)				
No (*n* = 255)	235 (94.8)	20 (64.5)	< 0.001	Chi-square
Yes (*n* = 24)	13 (5.2)	11 (35.5)		
Meningitis (*n* = 273)				
No (*n* = 272)	245 (99.6)	27 (100)	0.901	Fisher's exact
Yes (*n* = 1)	1 (0.4)	0 (0.0)		
Others (*n* = 273)				
No (*n* = 187)	164 (66.7)	23 (85.2)	0.035	Fisher's exact
Yes (*n* = 86)	82 (33.3)	4 (14.8)		

⁣^∗^Chi-square test.

⁣^∗∗^Fisher's exact test.

**Table 11:** Management of diabetic ketoacidosis.

Management aspect	Frequency	Percentage (%)
Fluids started within 1 h of presentation (*n* = 281)		
No	3	1.1
Yes	278	98.9
Started insulin units per kg/h (*n* = 275)		
≤ 5	168	61.1
> 5	107	38.9
Antibiotics (*n* = 279)		
No	109	39.1
Yes	170	60.9
Enoxaparin (*n* = 281)		
No	160	56.9
Yes	121	43.1
Outcome (*n* = 257)		
Discharge	234	91.0
Discharge against medical advice	21	8.2
Transfer to another hospital	2	0.8
Death	0	0.0
Out of dehydration (h) (*n* = 241)		
≤ 6	217	90.0
> 6–12	24	10.0
Out of ketosis (h) (*n* = 262)		
Within 24	246	93.9
> 24	16	6.1
Out of hospital (h) (*n* = 254)		
≤ 24	139	54.7
24–48	81	31.9
72	17	6.7
> 72	17	6.7

**Table 12:** Comparison of diabetic ketoacidosis management between Type 1 and Type 2 diabetes mellitus patients.

Management aspect	Type 1 diabetes *N* (%)	Type 2 diabetes *N* (%)	*p* value
Fluids started within 1 h of presentation (*n* = 281)			
No (*n* = 3)	2 (0.8)	1 (3.1)	0.305⁣^∗∗^
Yes (*n* = 278)	247 (99.2)	31 (96.9)	
Started insulin units per kg/h (*n* = 275)			
≤ 5 (*n* = 168)	164 (66.7)	4 (13.8)	< 0.001⁣^∗∗^
> 5 (*n* = 107)	82 (33.3)	25 (86.2)	
Antibiotics (*n* = 279)			
No (*n* = 109)	99 (40.1)	10 (31.3)	0.335⁣^∗^
Yes (*n* = 170)	148 (59.9)	22 (68.8)	
Enoxaparin (*n* = 281)			
No (*n* = 160)	155 (62.2)	5 (15.6)	< 0.001⁣^∗^
Yes (*n* = 121)	94 (37.8)	27 (84.4)	
Outcome (*n* = 257)			
Discharge (*n* = 234)	212 (91.0)	22 (91.6)	0.109⁣^∗^
Discharge against medical advice (*n* = 21)	20 (8.6)	1 (4.2)	
Transfer to another hospital (*n* = 2)	1 (0.4)	1 (4.2)	
Out of dehydration (h) (*n* = 241)			
≤ 6 (*n* = 217)	197 (90.4)	20 (87.0)	0.408⁣^∗∗^
> 6–12 (*n* = 24)	21 (9.6)	3 (13.0)	
Out of ketosis (h) (*n* = 262)			
Within 24 (*n* = 246)	220 (93.6)	26 (96.3)	0.492⁣^∗∗^
> 24 (*n* = 16)	15 (6.4)	1 (3.7)	
Out of hospital (h) (*n* = 254)			
≤ 24 (*n* = 139)	122 (54.0)	17 (60.7)	0.854⁣^∗^
24–48 (*n* = 81)	73 (32.3)	8 (28.6)	
72 (*n* = 17)	16 (7.1)	1 (3.6)	
> 72 (*n* = 17)	15 (6.6)	2 (7.1)	

⁣^∗^Chi-square test.

⁣^∗∗^Fisher's exact test.

## Data Availability

The data supporting the findings of this study are available from the corresponding author upon reasonable request. Restrictions apply to the availability of these data, which were used under license for the current study, so they are not publicly available. However, the data may be made available by the authors upon reasonable request and with permission from the institution/organization, if applicable.
